# Study on High Frequency Surface Discharge Characteristics of SiO_2_ Modified Polyimide Film

**DOI:** 10.3390/polym13244387

**Published:** 2021-12-14

**Authors:** Zhaoliang Xing, Wenhan Chen, Zhihui Li, Naifan Xue, Fei Li, Xiying Dai, Shaowei Guo, Huize Cui

**Affiliations:** 1 State Key Laboratory of Advanced Power Transmission Technology, Global Energy Interconnection Research Institute Co., Ltd., Beijing 102209, China; 15811444029@163.com (Z.X.); lifei_geiri@163.com (F.L.); daixiying311@163.com (X.D.); m18826072964@163.com (S.G.); jameschz@126.com (H.C.); 2 State Key Laboratory of Alternate Electrical Power System with Renewable Energy Sources, North China Electric Power University, Beijing 102206, China; nev777@ncepu.edu.cn; 3 State Grid Zhejiang Electric Power Co., Ltd., Construction Company, Hangzhou 318016, China; 15968818567@163.com

**Keywords:** polyimide, nano-SiO_2_, life time, high-frequency electrical stress, creeping development

## Abstract

Polyimide (PI) can be used as a cladding insulation for high frequency power transformers, and along-side discharge can lead to insulation failure, so material modification techniques are used. In this paper, different doped nano-SiO_2_ are introduced into polyimide for nanocomposite modification. The results of testing the life time of high-frequency electrical stress along-side discharge show that the 10% SiO_2_ doping has the longest life time. The results show that: for composites prone to corona, their flashover causes more damage, and both positive half-cycle and polarity reversal discharges are more violent; compared to pure PI, the positive half-cycle and overall discharge amplitude and number of modified films are smaller, but the negative half-cycle is larger; at creeping development stages, the number of discharges is smaller, and the discharge amplitude of both films fluctuates in the mid-term, with the modified films having fewer discharges and the PI films discharging more violently in the later stages. The increase in the intensity of the discharge was greater in the later stages, and the amplitude and number of discharges were much higher than those of the modified film, which led to a rapid breakdown of the pure polyimide film. Further research found that resistivity plays an important role in the structural properties of the material in the middle and late stages, light energy absorption in the modified film plays an important role, the distribution of traps also affects the discharge process, and in the late stages of the discharge, the heating of the material itself has a greater impact on the breakdown, so the pure polyimide film as a whole discharges more severely and has the shortest life.

## 1. Introduction

High Frequency Power Transformer (HFPT) with highly controllable primary and secondary voltages and currents for high power quality. As one of the best insulating organic materials, Polyimide (PI) is often used for interturn insulation of HFPT [1]. The high frequency sine wave inside the HFPT will aggravate the skin effect and proximity effect of the ferromagnetic material inside the device, leading to a significant increase in insulation loss [2,3] and the temperature rise. At the same time, the flashover field strength along the surface is much smaller than the air gap breakdown field strength of the same gap, so creeping discharge is prone to occur at the HFPT tip defect, which will eventually cause insulation failure [4]. Therefore, it is essential to study the high frequency creeping discharge of polyimide.

Previous studies have shown that nano-composite modification of polyimide can effectively improve its comprehensive properties including corona resistance, heat resistance [5,6,7], and the doping of silicon-containing particles in polyimide can improve the comprehensive properties of the material, including anti-atomic oxygen performance, dielectric property [8,9]. However, there is a lack of research on inter-turn insulation of high-frequency transformers, that is, the development process and characteristics of high-frequency creeping discharge of polyimide film under extremely uneven fields, especially after the polyimide is modified by nanocomposite with SiO_2_.

For this reason, this article uses in-situ polymerization to prepare modified polyimide films doped with nano-SiO_2_ particles containing 2%, 4%, 6%, 8%, and 10% by mole content of Si. First, we test the high-frequency creeping discharge life, and then the longest-life modified film and pure polyimide film are used to observe the development process of creeping discharge and collect discharge data, combining ultraviolet-visible spectroscopy, scanning electron microscopy (SEM), crystallinity, resistivity, and dielectric properties tests. The reasons for the increase in electrical life are studied and analyzed, and we provide a theoretical basis for the modification and selection of high-frequency inter-turn insulation materials.

## 2. Materials and Methods

In this paper, we used a two-step method to prepare the polyimide film [10], taking N,N-dimethyl-acetamide (DMAC) as a polar solvent and adding 4,4-diaminodiphenyl ether (ODA) diamine monomer and the nano SiO_2_ particles with a particle size of 15 ± 5 nm. After fully dissolving and dispersing, 1.02 times the molar content of dianhydride monomer of diamine known as the pyromellitic anhydride (PMDA) were added in a ratio of 6:3:1 every half an hour and mild mechanical stirring was performed under constant temperature conditions. After 12 h, a viscous golden yellow polyamide acid solution (PAA) solution was obtained, and then vacuuming and coating were performed. Finally, a golden polyimide film with better quality was obtained by stepwise heating. The films are named as Si0, Si2, Si4, Si6, Si8 and Si10 according to the content of SiO_2_ particles added. The sources and purity information of related chemical reagents are shown in the following Table 1.

**Figure 1:**
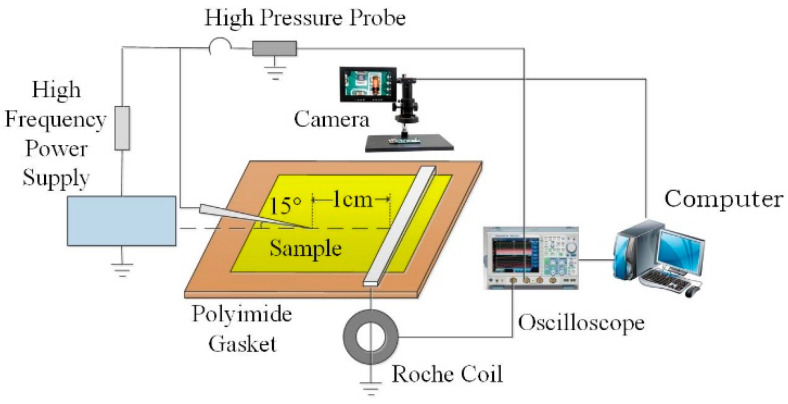
High frequency creeping discharge platform.

The high frequency surface discharge platform is shown in Figure 1 below. The power supply is high frequency and high voltage sinusoidal power supply, and the voltage frequency is continuously adjustable in the range of 5~50 kHz. The needle-plate electrode was used to simulate the discharge defect along the surface of the tip in the high-frequency power transformer, and the angle between the needle and the horizontal plane was 15°. The needle electrode was 10 mm away from the plate electrode, and the sinusoidal voltage of 11 kV and 20 kHz was applied to the needle electrode. Real-time measurement of applied voltage changes with Tektronix P6015A high-voltage probe with a decay factor of 1000:1. KH-100 pulse current sensor with high sensitivity was used to collect discharge data along the electrode surface. Before the test, the film needs to be wiped and dried with anhydrous ethanol. In order to ensure the reliability of the experimental results, 5 samples of each doping system were taken for 10 times of life experiments to obtain the mean value.

The time from the appearance of corona on the film surface to the eventual occurrence of surface flashover is recorded as the material life. The statistical life of five films is obtained by using the Weibull statistical method as shown in Table 2 [11]. It was found that after doping SiO_2_ particles, the lifetime increased with the increase of doping content. Among them, Si0 has the shortest lifetime and Si10 has the longest lifetime, which is 3.40 times that of pure polyimide. Therefore, in order to further study the effect of nano-composite modification of doped nanoparticles on the surface discharge characteristics of polyimide, the more representative Si0 and Si10 were photographed, and the surface discharge data were collected.

## 3. Experimental Results

### 3.1. Influence Mechanism of Absorption of Light Energy

**Figure 2:**
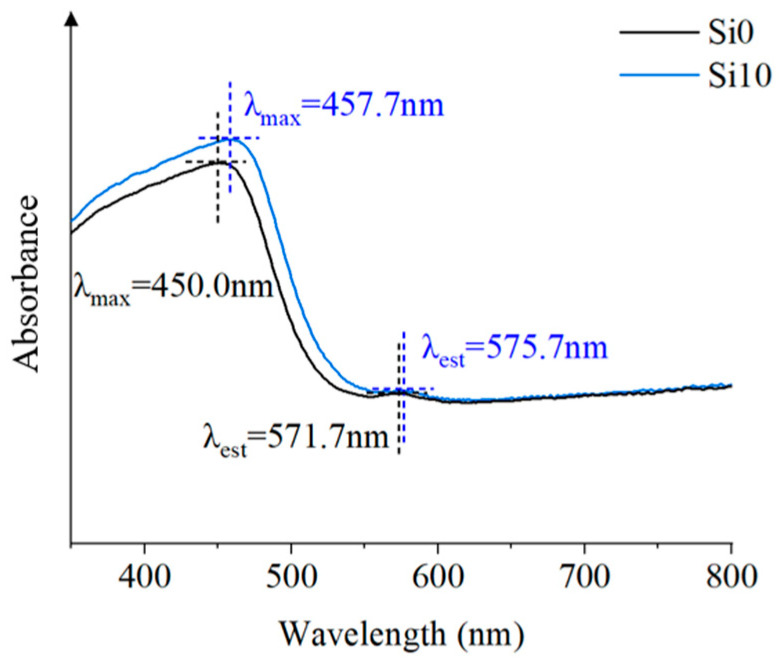
Ultraviolet-Visible Spectroscopy.

The light energy has a great influence on the discharge behavior of the material. Ultraviolet-visible spectroscopy can test the absorption characteristics of light energy and characterize the charge transfer complexation. The transmittance mode of Shimadzu UV-2600 ultraviolet and visible spectrophotometer (UV-Vis) can be directly used to test the absorbance of the film. The wavelength range of the test was 300–650 nm. According to the Ultraviolet-visible spectroscopy of Figure 2 in the wavelength range of 350–800 nm, the spectral characteristics can be characterized by the peak wavelength and the maximum absorption wavelength. Among them, the peak wavelength of Si10 is larger, and the absorption of Si10 is red shifted from the maximum absorption wavelength. After the addition of SiO_2_ nanoparticles, the maximum absorption wavelength in the near-UV region and the visible region moves to the long wave direction, namely, the red shift occurs. This indicates that after the introduction of SiO_2_, SiO_2_ is more likely to absorb light energy, easily form intermolecular electron cloud conjugation, increase the charge transfer complexation of polyimide, and enhance the intermolecular force of the material to improve the compactness and discharge tolerance of the composite film. For polymers, the red shift of the curve also indicates that the increase of the conjugated system improves the compactness and regularity of the materials, which will effectively hinder the movement of carriers, delay the formation of conductive channels, and be more difficult to form electronic collapse, thereby improving the insulation performance [12]. Therefore, the overall average discharge amplitude and discharge times per unit time of Si10 are lower, with the less energy collected by the plate electrode. By contrast, the energy collected by the Si0 plate electrode is the most.

### 3.2. Influence Mechanism of Material Structure

**Figure 3:**
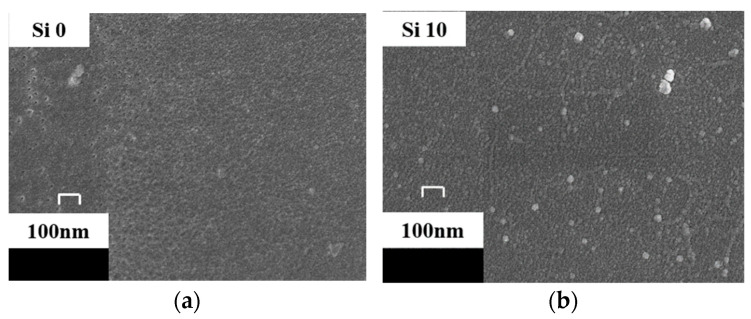
Surface morphology before electrical aging. (**a**) Si0. (**b**) Si10.

**Figure 4:**
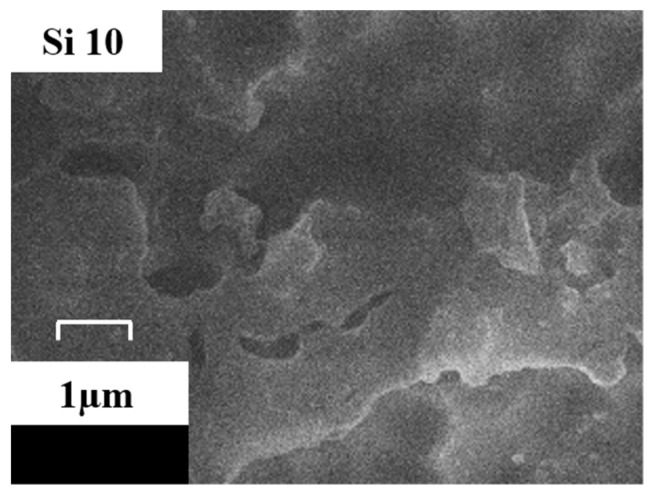
Surface morphology after electrical aging.

The material structure directly affects the characteristics of creeping discharge signals, characterization of Surface Morphology of Si0 and Si10 after gold spraying by scanning electron microscope (SEM, HITACHI S4800), so observe the surface morphology of Au-sprayed Si0 and Si10 with 40,000 times magnification, as shown in Figure 3. It is found that there is an amorphous region on the surface of pure polyimide, which is due to the semicrystalline nature of polyimide itself. After doping 10% SiO_2_ particles, brighter nanoparticles are distributed on the surface, and the surface of the matrix is relatively regular, which is consistent with the results of ultraviolet-visible spectroscopy. This is because after nano-composite modification, the hydroxyl groups on the surface of nanoparticles form hydrogen bonds during in-situ polymerization, which are linked to polyimide molecules. Thus, the inorganic/organic interface is formed in the polyimide nano-composite system. According to the multi-regional structure model [13], the interface between inorganic particles and matrix makes the molecular chains in the transition region orderly arranged, and the force is more average under electrical stress. At the same time, SEM is used to observe the Si10 surface after 40 min electrical aging. As shown in Figure 4, it’s Si10, after 40 min of discharge, SiO_2_ particles are exposed to form a blocky and bright inorganic phase, which will play a role in scattering electrons and reducing electron kinetic energy. Therefore, after adding nanoparticle SiO_2_, compared with pure polyimide, the interface effect and the exposed inorganic phase can reduce the discharge amplitude and discharge times overall.

At the same time, the introduction of inorganic phase in the material also inevitably causes defects, resulting in increasing the internal trap of the material. The trap is essentially a local state that can capture the charge. When the applied electric field energy exceeds the trap barrier, the charge enters the trap, and the charge after sinking is still likely to collapse. If the energy obtained is not enough to cross the trap barrier, the charge will accumulate in the trap to form a stable space charge, which will affect the local electric field. The sinking, collapsing and other behaviors of charges in it have a greater impact on the discharge behavior of the material surface. According to the bipolar carrier model [14], nanocomposite modification increases traps by introducing physical defects. In the discharge process, the traps will capture the charge and form a reverse electric field, which plays a role in homogenizing the electric field. Therefore, in general, the Si10 plate electrode has the least energy collection and lower discharge level, which also leads to the increase of the life of the modified film.

### 3.3. Development Morphology of High Frequency Creeping Discharge

**Figure 5:**
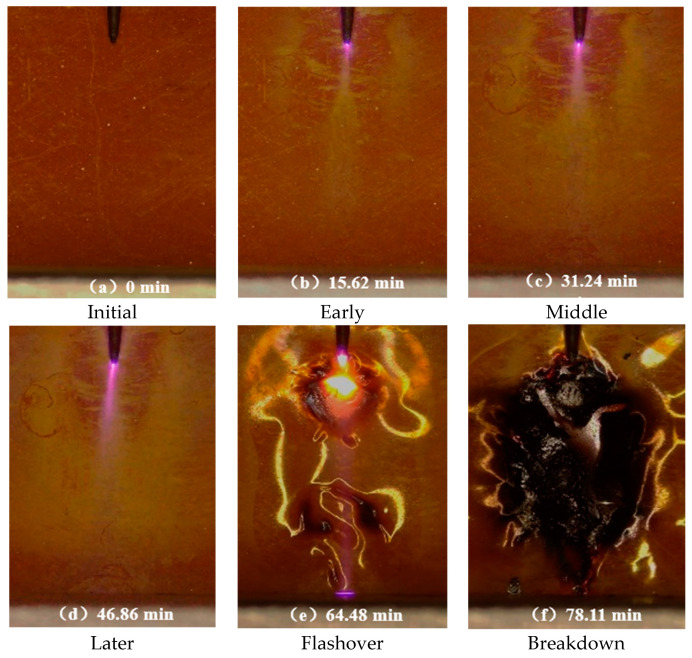
(**a**–**f**) The scatter plots for different discharge time of Si10 film.

Different from the corona generated by the needle electrode under power frequency, it is not easy to move forward and easy to longitudinal breakdown. At high frequency, the corona is easy to move forward and flashover occurs, and the film damage is more serious. The corona development morphology of Si0 and Si10 is basically the same as the macro morphology of the film surface, as shown in Figure 5. At the junction of the tip and the material, a faint blue-violet halo first appeared, accompanied by a slight discharge noise. At this time, on the surface of the film, a forward and diffuse spindle-shaped white spot appeared ahead of the tip, and an uneven white spot spread outward around the tip. The white spot on the surface eroded by electric stress was formed due to the surface degradation of the material and the dissociation of the adsorbed gas and water. With the development of surface discharge, the corona extends forward, the corona light closes to the material, the white spot area expands and the color deepens; in the late discharge period, the corona swings, even in the absence of light environment can be observed that the corona tip occasionally touch the ground electrode, which is prone to flashover; in the flashover stage, the streamer formed in the extremely uneven field is unstable, and the movement rate of the charge under the high frequency voltage is much higher than that of the power frequency. Therefore, when the front end of the streamer reaches the plate electrode, there may be a left and right deviation of the penetrating arc channel between the needle and the plate electrode, and obvious deformation caused by thermal stress and electrical stress on the surface of the material. In the breakdown stage, breakdown occurs under high-frequency electrical stress, resulting in complete damage to the material and even combustion on the surface. Therefore, the damage degree of power frequency electrical stress to the material is far less than that of high-frequency electrical stress [15].

It is worth noting that after normalization treatment, the development of Si10 corona is significantly faster at the early stage of discharge. The white spot length of Si10 is the longest after observing the material surface after discharge for 5 min. However, at the later stage of discharge, the development of Si0 corona was significantly accelerated, and the white spot area on the surface was relatively large, and the rapid flashover led to breakdown.

### 3.4. Acquisition and Analysis of High Frequency Creeping Discharge Signal

**Figure 6:**
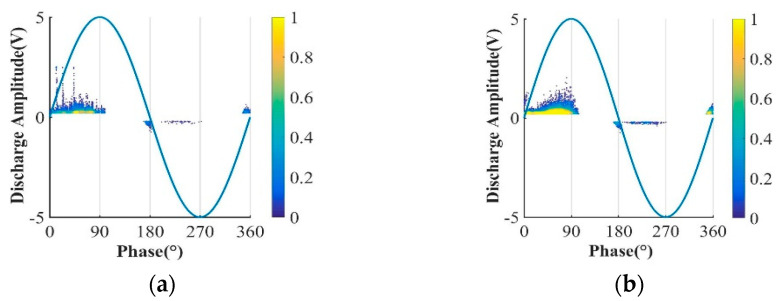
Surface discharge spectrum. (**a**) The creeping discharge spectra of the Si0. (**b**) The creeping discharge spectra of the Si10.

The discharge signals collected by the ground electrode are de-noised and counted. The creeping discharge spectra of the two films during their lifetime are shown in Figure 6.

As shown in the figure above, under high frequency voltage, the positive half-cycle discharge amplitude and discharge times are significantly greater than the negative half-cycle, and the discharge will be relatively violent at the voltage polarity reversal, which is caused by the negative charge and the same polarity charge accumulated on the surface of material at high frequency. The discharge spectra of the positive half cycle of the three films are different. The Si0 discharge is more dispersed, and a high amplitude creeping discharge pattern appears. The pattern of Si10 is flatter and the overall amplitude is lower. Quantitative statistics of the average discharge amplitude and the number of discharges per unit time of the two films are shown in Table 3.

The quantitative statistical results once again confirmed that the positive half cycle discharge amplitude and discharge times of the two films are greater than the negative half cycle, and the average discharge amplitude and discharge times of the Si10 overall and positive half cycle are smaller, which is consistent with the law of the life of them, and the law of negative half cycle is just the opposite.

**Figure 7:**
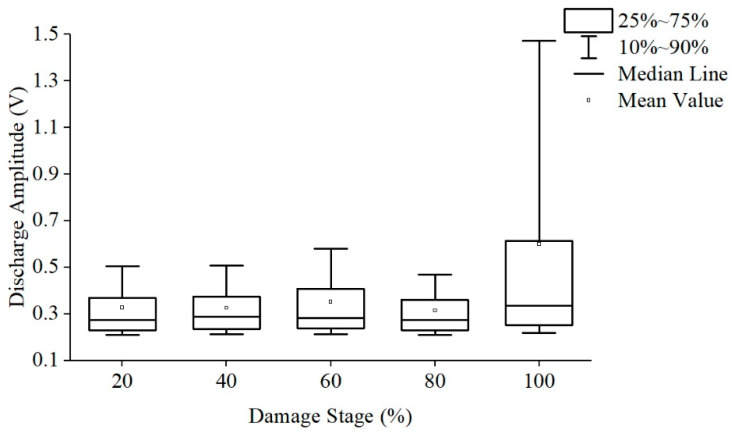
The discharge amplitude of Si0 at different stages.

**Figure 8:**
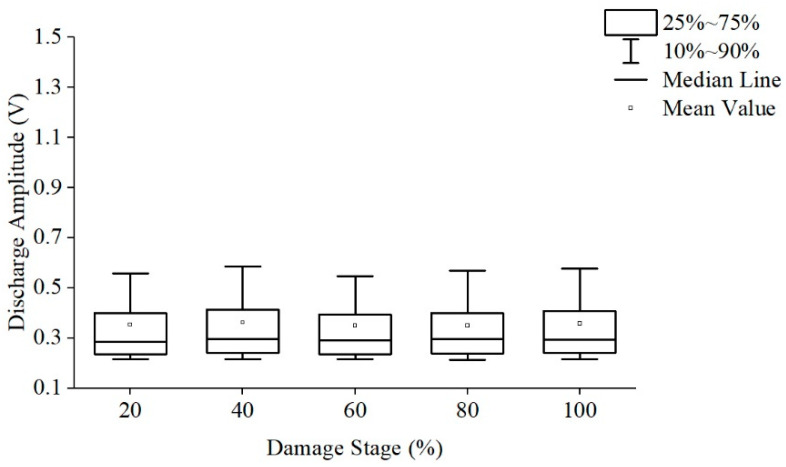
The discharge amplitude of Si10 at different stages.

**Figure 9:**
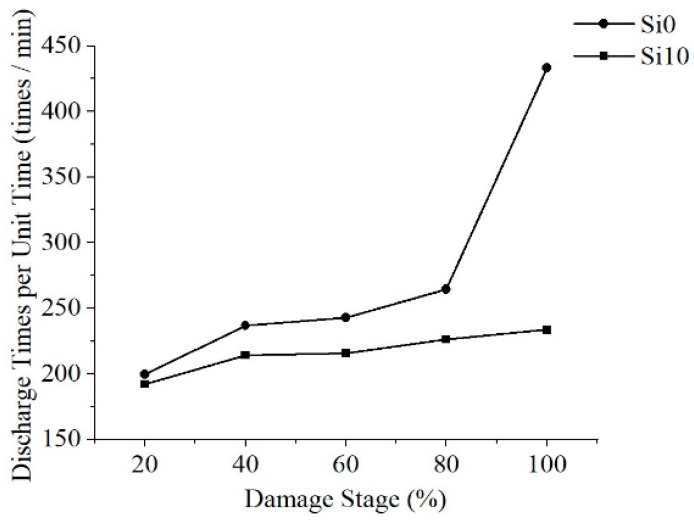
Average discharge times at different stages.

According to the characteristics of creeping discharge, the creeping discharge process is normalized and divided into five stages and count the discharge amplitude and average number of discharges under each stage. The discharge amplitudes are shown in Figure 7 and Figure 8, respectively. The average discharge times of discharges is shown in Figure 9.

The development of the discharge amplitude and average number of discharges of the two films has regularity: As the damage stage increases, the discharge amplitude fluctuates, reaching the highest value in the full life in the last stage, and the number of discharges per unit time continues to rise and reaches its peak in the last stage. This rule has nothing to do with whether polyimide is modified or not, the last stage of discharge amplitude increase may be one cause of polyimide final flashover breakdown, the continuous increase of discharge times per unit time will aggravate the damage degree of the film, so the discharge amplitude can be used as the signal of its adjacent flashover and the basis of material damage, and the discharge times per unit time can be used as the basis to judge the damage degree of material.

The development of discharge amplitude and discharge times is also different: the discharge amplitude of Si10 is large in the early stage, and Si10 is close to Si0 in discharge times, while the discharge amplitude of Si0 increases greatly and far exceeds Si10 in the late stage, and the discharge times increase more, thus far exceed Si10 level.

In conclusion, especially in the middle and late stage of discharge, large discharge amplitude and more discharge times will aggravate the damage of the insulation performance along the surface, resulting in the life of Si0 is much lower than that of Si10.

## 4. Discussion on Influence Mechanism

The secondary electron emission avalanche (SEEA) model can be used to explain its development morphology [16]. Effective initial electrons are generated at the three junction points of needle electrode, air, and polyimide. Under the action of strong field, collision ionization occurs and gradually forms electron avalanche. The development of electron avalanche makes the discharge enter the streamer discharge stage. The strong electric field at the head of the streamer is prone to excitation. When it returns to the normal state, photons will be emitted. Positive and negative particles combine easily under the action of electric field inside electron avalanche, accompanied by a lot of photons, which promotes the spatial photoionization and the photoinduced electron emission of polyimide. Under the extremely uneven field of needle-plate, the tangential electric field is strengthened. The streamer develops in a linear form until the ion area reaches the ground electrode, forming a penetrating conductive channel, which results in arc discharge.

In order to further study the influence mechanism of nano-SiO_2_ doping on high frequency surface discharge, the physical and chemical properties of Si0 and Si10 were tested, including resistivity and dielectric properties, and the influence of modification on high frequency surface discharge characteristics was analyzed from different angles.

### 4.1. Influence Mechanism of Carrier Migration

The development of creeping discharge is determined by the movement of carrier migration on material surface, and resistivity directly affects the movement characteristics of carrier [17]. The volume resistivity and surface resistivity of the films are measured by using the 6517B high resistance meter and the 8009 three-electrode fixture made in the United States, the results are shown in Table 4. The resistivity test demonstrates that the doping of SiO_2_ particles reduces the volume resistivity and surface resistivity of the material, which is caused by the introduction of impurity ions to increase the number of carriers.

For the resistivity, the change of resistivity effects the movement of charged particles on the surface of the material: on the one hand, the electrons play a major role in the development of creeping discharge, with low resistivity and short time of electrons passing through the gap, resulting in the electron avalanche current to be larger [18]. On the other hand, the space charge affects the characteristics of high frequency creeping discharge. The space charge diffuses along the surface of the material under the tangential electric field, and the resistivity is low, which leads to the easy dissipation of space charge and the increase of discharge amplitude and frequency. Therefore, for Si10, especially in the early stage, the discharge amplitude and frequency are larger, and there are more local intensive discharges, so the white spots develop faster.

### 4.2. Influence Mechanism of Dielectric Properties

**Figure 10:**
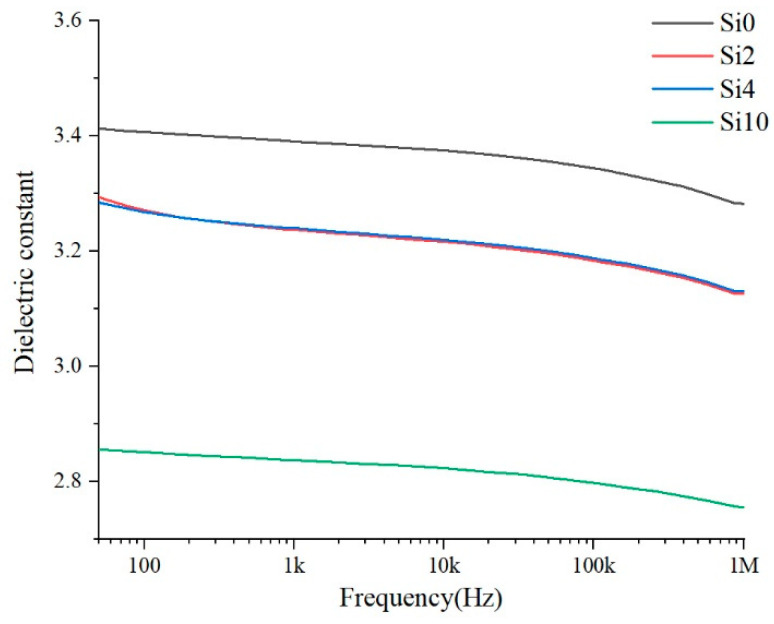
Relative dielectric constant.

**Figure 11:**
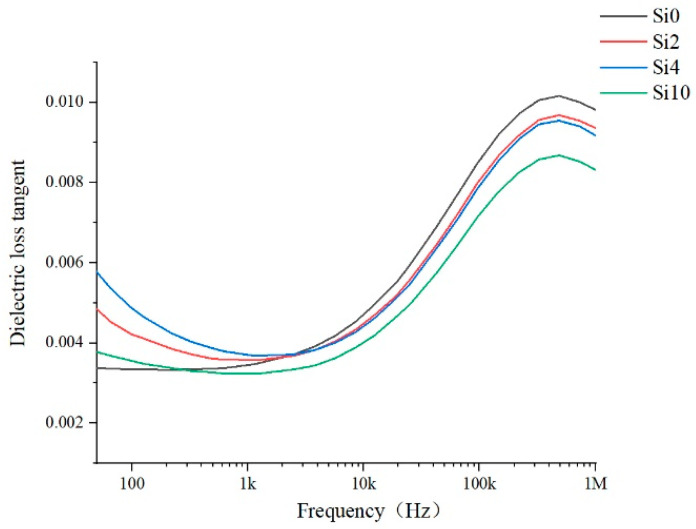
Dielectric loss tangent.

The dielectric properties of the materials characterize the polarization properties of polymer materials and the loss under alternating electric field. More heat is generated under high-frequency stress, which affects the high-frequency surface discharge life. The dielectric properties of the samples Si0, Si2, Si4 and Si10 are tested and the relative permittivity and dielectric loss tangent values are shown in Figure 10 and Figure 11.

For relative dielectric constant, as the Figure 10 shown, in the range of 50 Hz–1 MHz, the relative dielectric constant will decrease after adding nano-SiO_2_ particles, and the more the content is added, the more obviously the relative dielectric constant decreases. This is because SiO_2_, as an atomic crystal, has a lower dielectric constant than that of polyimide. At the same time, the addition of inorganic particles generates a two-phase interface, which inevitably leads to the introduction of air gap [19] and the relative dielectric constant of air is 1. Therefore, the more the content added, the higher the relative dielectric constant of the composite is.

For dielectric loss tangent, it can be seen from the Figure 11 that dielectric loss tangent of Si0 is the lowest in the low frequency range below 1 kHz, while that of the modified film is relatively high. But in the high frequency range, especially above 10 kHz, the dielectric loss tangent of Si0 is the highest, while the modified film is relatively lower, and the dielectric loss of Si10 is the lowest. The reason is that in the low frequency region, due to the addition of nanoparticles, the material inevitably causes more impurity ions and so on, which will lead to more polarization loss in the alternating current field. However, in the high frequency range, the dielectric loss caused by the displacement polarization of impurity particles accounts for a small proportion. Polyimide is a weak polar dielectric, and the increase in frequency leads to the relaxation polarization of polar groups. Therefore, the loss is caused by the intermolecular force, and the proportion of this loss is larger. In this case, the addition of SiO_2_ reduces the number of polar groups in the same volume of composites, and the free volume of molecules is also larger, thereby reducing the dielectric loss.

### 4.3. Influence Mechanism of Dielectric Properties

From the analysis results of the representative physical and chemical properties of the above part, it can be seen that some basic characteristics of polyimide materials have changed significantly due to nano-composite modification. These changes in physical and chemical properties will cause different characteristics of materials in different discharge stages, and the influence mechanisms are also different in different stages.

It can be seen from the analysis that at the early stage of discharge, the material is in the development stage of electron avalanche, and the film surface is relatively complete at this time, so it is greatly affected by the resistivity. The low resistivity of Si10 is beneficial to movement of electron and promoting collision ionization, at the same time, the Si10 charge dissipates quickly, which further mitigates the inhibitory effect of homopolar charges that are less than dissipated at high frequencies on this half-cycle discharge, thus accelerating the development of electron avalanches. Therefore, Si10 has the fastest development in the early stage, and the discharge amplitude is relatively larger, and the number of discharges is similar to that of Si0. The negative charge accumulated on the surface of Si10 is less, which causes the negative half cycle discharge to be more intense.

In the middle and late stages of discharge, the surface of the material is basically degraded, and the structural characteristics and light absorption characteristics of the material are more important. The results of SEM shows that the inorganic particles are uniformly distributed in the matrix, and they will form an interface with the matrix of the polymer. On the one hand, the inner layer of the interface will capture the charge first under electrical stress, and the inner layer material has stronger ability to withstand electrical stress and is first destroyed, thus reducing the damage caused by charged particles on the material itself. On the other hand, polyimide is a polymer with long-range disorder and short-range order. The matrix molecular chain arrangement of the outer layer of the interface is more uniform and orderly, which helps the matrix to withstand electrical stress uniformly. In the middle and late discharge period, observe the surface morphology of the material after electrical aging, the film in the corona process is degraded by impacts of high temperature ablation, free radical reaction and charged particle and so on, and the nano-SiO_2_ particles in the surface doping layer are gradually deposited and formed a protective layer of massive nanoparticles, which could effectively prevent the damage of corona discharge on the substrate [20]. When it comes to light absorption characteristics, according to the ultraviolet-visible spectrum, Si10 has stronger ability to absorb light energy to form charge transfer complexation. For one hand, it not only absorbs more light energy, but it is a key stage for the development of discharge streamer during the middle and late discharge period. The absorption of light energy will reduce the promoting effect of light energy on space photoionization and photoinduced electron emission [21]. For another, light energy is used to form the charge transfer complexation between molecules, increase the conjugated system, enhance the intermolecular force, and further improve its resistance of high-frequency creeping discharge. At the same time, the distribution of the number of material traps also has a certain impact. In essence, the trap is a local state that can capture the charge, when the energy of the applied electric field exceeds the trap barrier, the charge enters the trap, and the charge after entering the trap is still likely to collapse. If the energy obtained is not enough to cross the trap barrier, the charge will accumulate in the trap to form a stable space charge, thus affecting the local electric field. In the middle and late stages of discharge, the material traps are basically filled with enough charges. There are more traps on the surface of Si10 film, and more charges are captured. On the one hand, this reduces the migration of charges to inhibit the damage of creeping discharge on the material. On the other hand, the space charge is more beneficial to the homogenization of the electric field.

The later stage of discharge is the period that material is near to be destroyed. At this time, the resistance and life of the material are not only determined by electrical stress, but also by the thermal stress accumulated in the previous stage of discharge. Thermal stress will destroy the integrity of polymer molecules and the arrangement and distribution of molecules. Excessive temperature will change the material from glass state to high elastic state, thereby reducing the resistance of the material to electrical stress, and ultimately causing electrical-thermal stress aging breakdown. Therefore, the high-frequency creeping discharge will cause severe combustion on the material surface during the flashover breakdown stage. At this time, the heat caused by the dielectric loss of the material is more important. Compared with pure polyimide, the tangent value of the dielectric loss angle of Si10 is lower, so the heat generated by the material under the alternating electric field in unit time is less, which reduces the damage of thermal stress to the material structure.

## 5. Conclusions

In this paper, the polyimide films are respectively doped with nano-SiO2 particles containing 2%, 4%, 6%, 8% and 10% by mole content of Si, and tested with pure polyimide film Si0.Obtain the following conclusions:

(1) After the doped nano-SiO_2_ particles are modified by nano-composite, the high-frequency creeping discharge life of polyimide increases with the increase of doping content, and the life of Si10 is the longest, which is 3.40 times that of pure polyimide Si0.
(2) In the process of discharge development, compared with the power frequency, both of the coronas of Si0 and Si10 are more likely to develop forward, and the flashover causes greater damage to the film. Both of them show more intense discharge at the positive half cycle and polarity reversal. However, in the development of corona and film white spot, Si10 is faster than Si0.
(3) In terms of the characteristics of high-frequency creeping discharge signals, for the positive half cycle and the whole, the discharge amplitude and discharge times per unit time of Si10 are lower than those of Si0, and the negative half cycle is opposite. The discharge amplitudes of the two films fluctuated first and then increased to the peak, and the discharge times per unit time continued to increase. In the early stage, the discharge amplitude of Si10 is large, and the number of discharge is close to that of Si0. However, in the middle and late stages, Si0 develops rapidly, which is significantly larger than that of Si10, resulting in rapid breakdown.
(4) The influence mechanism of nano-composite modification on life is revealed as the following:

In the early stage, resistivity has a great influence on its life that the smaller the resistivity, the faster the development of surface discharge. And in the middle and late stages, the structural characteristics, light absorption characteristics and trap distribution of the material play vital roles. The effect of interface of Si10 and absorbed light energy will reduce the damage of creeping discharge on the film, and the trap filled with charge will homogenize the electric field. At the later stage of discharge, the dielectric loss of Si10 is smaller, which also inhibits the electrical-thermal aging of the material.

## Figures and Tables

**Table 1:** Chemical reagents for preparation of Polyimide films.

Name	Purity	Supplier
Pyromellitic dianhydride	99%	Shanghai Macklin Biochemical Co., Ltd., Shanghai, China
4,4′-Diaminodiphenyl ether	98%	Shanghai Macklin Biochemical Co., Ltd., Shanghai, China
4,4′-thiobisbenzenamine	99%	Jiangxi Renming Pharmaceutical Chemical Co., Ltd., Jiangxi, China
N,N-Dimethylacetamide	99%	Sinopharm Chemical Reagent Co., Ltd., Shanghai, China

**Table 2:** Statistical life of creeping discharge.

Type	Si0	Si2	Si4	Si6	Si8	Si10
Life/min	23.12	42.40	45.40	53.50	69.11	78.11

**Table 3:** Quantitative statistics of creeping discharge.

Type		Si0	Si10
Average	Overall	0.399	0.354
discharge	Positive half week	0.407	0.359
amplitude	Negative half week	0.288	0.302
Average	Overall	267.67	216.35
discharge	Positive half week	249.59	197.35
times	Negative half week	18.08	19

**Table 4:** The volume resistivity and surface resistivity.

Type	Si0	Si10
volume resistivity (10^13^ Ω/m)	7.838	6.554
surface resistivity (10^10^ Ω/m)	2.613	2.5

## Data Availability

The data presented in this study are available on request from the corresponding author.

## References

[1] Kim G., Choi M., Lee D., Ha C. (2012). 2D-aligned graphene sheets in transparent polyimide/graphene nanocomposite films based on noncovalent interactions between poly (amic acid) and graphene carboxylic acid. Macromol. Mater. Eng..

[2] Aggeler D., Biela J., Kolar J. A compact, high voltage 25 kW, 50 kHz DC-DC converter based on SiC JFETs. Proceedings of the IEEE Applied Power Electronics Conference and Exposition.

[3] Le Besnerais J., Fasquelle A., Hecquet M., Pelle J., Lanfranchi V., Harmand S., Brochet P., Randria A. (2010). Multiphysics Modeling: Electro-Vibro-Acoustics and Heat Transfer of PWM-Fed Induction Machines. IEEE Trans. Ind. Electron..

[4] Du S., Baek Y., Wang G., Bhattacharya S. Design Considerations of High Voltage and High Frequency Transformer for Solid State Transformer Application. Proceedings of the IECON 2010—36th Annual Conference on IEEE Industrial Electronics Society.

[5] Yi H., Qin J., Yi G. (2004). Polyimide–silica hybrid films made from polyamic acids containing phenolic hydroxyl groups. J. Appl. Polym. Sci..

[6] Liu L., Shi H., Weng L., Ding J., Cui W. (2014). The effects of particle size on the morphology and properties of polyimide/nano-Al_2_O_3_ composite films. Polym. Polym. Compos..

[7] Zha J., Song H., Dang Z., Shi C., Bai J. (2008). Mechanism analysis of improved corona-resistant characteristic in polyimide/TiO_2_ nanohybrid films. Appl. Phys. Lett..

[8] Vernigorov K., AYAlent’Ev Muzafarov A., Novikov L., Chernik V. (2011). Erosion of polyimide modified by amorphous silica sol in the stream of oxygen plasma. J. Surf. Investig..

[9] Li J., Xue B., Yang H., Liu L., Xie H., Pei Y., Lu P., Wang G., Wang J., Li J. Fabrication of LED Full-Color Display Matrix with Small Pixel. Proceedings of the Fourteenth International Conference on Solid State Lighting and LED-Based Illumination Systems.

[10] Kim K., Kim H., Kim M., Kim H., Choi S., Kim S. (2009). Two-step polyimide curing technique for flexible plastic liquid crystal devices. JPN J. Appl. Phys..

[11] Zhou Q., Huang X., Wei B., Le L. (2021). Impulse Life Evaluation Method of MOV Based on Weibull Distribution. IEEE Access.

[12] Huang X., Wang J., Li Q., Lin J., Wang Z. (2019). Impact of the phenyl thioether contents on the high frequency dielectric loss characteristics of the modified polyimide films. Surf. Coat. Technol..

[13] Li S., Yin G., Chen G., Li J., Bai S., Zhong L., Zhang Y., Lei Q. (2010). Short-term breakdown and long-term failure in nanodielectrics: A review. IEEE Trans. Dielectr. Electr. Insul..

[14] Min D., Li S., Ohki Y. (2016). Numerical simulation on molecular displacement and DC breakdown of LDPE. IEEE Trans. Dielectr. Electr. Insul..

[15] Zhang K., Zhang L., Li Z., Zhao T., Zou L. (2019). Analysis of the Phenomena and Characteristics of Gas-Solid Insulation Surface Discharge under High Frequency Sinusoidal Electrical Stress. Trans. China Electrotech. Soc..

[16] Li Z., Xu H., Zheng X., Zhang L., Li S. (2020). Unraveling the transition from secondary electron emission dominated to surface charge trap dominated electronic avalanche process along the solid dielectric surface in vacuum. Appl. Phys. Lett..

[17] Lin H., Wang R., Xie Q., Zhang S., Shao T. (2017). Rapid surface modification by plasma jet to promote surface charge decaying. Trans. China Electrotech. Soc..

[18] Zhang X., Tang X., Guo Y., Liu K., Kang Y., Li Y., Wu G. (2019). Effect of High-speed Airflow on the Surface DC Discharge Characteristics of the Needle Plate. Proc. CSEE.

[19] Zhang Y., Lu S., Li Y., Dang Z., Xin J., Fu S., Li G., Guo R., Li L. (2005). Novel Silica Tube/Polymide Composite Films with Variable Low Dielectric Constant. Adv. Mater..

[20] Rogti F. (2011). Space charge dynamic at the physical interface in cross-linked polyethylene under DC field. IEEE Trans. Dielectr. Electr. Insul..

[21] Akyuz M., Gao L., Cooray V., Gustavsson T., Gubanski S. (2001). LarssonPositive streamer discharges along insulating surfaces. IEEE Trans. Dielectr. Electr. Insul..

